# Deep Learning-Based Detection of Carotid Plaques Informs Cardiovascular Risk Prediction and Reveals Genetic Drivers of Atherosclerosis

**DOI:** 10.1101/2024.10.17.24315675

**Published:** 2024-10-18

**Authors:** Murad Omarov, Lanyue Zhang, Saman Doroodgar Jorshery, Rainer Malik, Barnali Das, Tiffany R. Bellomo, Ulrich Mansmann, Martin J. Menten, Pradeep Natarajan, Martin Dichgans, Vineet K. Raghu, Christopher D. Anderson, Marios K. Georgakis

**Affiliations:** 1. Institute for Stroke and Dementia Research, LMU University Hospital, LMU Munich, Munich, Germany; 2. Program in Medical and Population Genetics and Cardiovascular Disease Initiative, Broad Institute of MIT and Harvard, Cambridge, MA, USA; 3. Cardiovascular Imaging Research Center, Massachusetts General Hospital, Harvard Medical School, Boston, MA, USA; 4. Division of Vascular and Endovascular Surgery, Massachusetts General Hospital, Boston, MA, USA; 5. Institute for Medical Information Processing, Biometry and Epidemiology, Medical Faculty, LMU Munich, Munich, Germany; 6. BioMedIA, Department of Computing, Imperial College London, London, United Kingdom; 7. Institute for AI in Healthcare and Medicine, School of Computation, Information and Technology, Technical University of Munich, Munich, Germany; 8. Cardiovascular Research Center and Center for Genomic Medicine, Massachusetts General Hospital, Boston, MA, USA; 9. Cardiovascular Disease Initiative, Broad Institute of Harvard and MIT, Cambridge, Boston, MA, USA; 10. Department of Medicine, Harvard Medical School, Boston, MA, USA; 11. Munich Cluster for Systems Neurology (SyNergy), Munich, Germany; 12. German Center for Neurodegenerative Diseases, (DZNE, Munich), Munich, Germany; 13. German Centre for Cardiovascular Research (DZHK, Munich), Munich, Germany; 14. McCance Center for Brain Health, Massachusetts General Hospital, Boston, MA, USA.; 15. Department of Neurology, Brigham and Women’s Hospital, Boston, MA, USA.

**Keywords:** atherosclerosis, carotid artery, vascular ultrasound, cardiovascular disease, machine learning, genetics, Mendelian Randomization

## Abstract

Atherosclerotic cardiovascular disease, the leading cause of global mortality, is driven by lipid accumulation and plaque formation within arterial walls. Carotid plaques, detectable via ultrasound, are a well-established marker of subclinical atherosclerosis. In this study, we trained a deep learning model to detect plaques in 177,757 carotid ultrasound images from 19,499 UK Biobank (UKB) participants (aged 47–83 years) to assess the prevalence, risk factors, prognostic significance, and genetic architecture of carotid atherosclerosis in a large population-based cohort. The model demonstrated high performance metrics with accuracy, sensitivity, specificity, and positive predictive value of 89.3%, 89.5%, 89.2%, and 82.9%, respectively, identifying carotid plaques in 45% of the population. Plaque presence and count were significantly associated with future cardiovascular events over a median follow-up period of up to 7 years, leading to improved risk reclassification beyond established clinical prediction models. A genome-wide association study (GWAS) meta-analysis of carotid plaques (29,790 cases, 36,847 controls) uncovered two novel genomic loci (p < 5×10^−8^) with downstream analyses implicating lipoprotein(a) and interleukin-6 signaling, both targets of investigational drugs in advanced clinical development. Observational and Mendelian randomization analyses showed associations between smoking, low-density-lipoprotein (LDL) cholesterol, and high blood pressure and the odds of carotid plaque presence. Our study underscores the potential of carotid plaque assessment for improving cardiovascular risk prediction, provides novel insights into the genetic basis of subclinical atherosclerosis, and offers a valuable resource for advancing atherosclerosis research at the population scale.

Atherosclerosis, characterized by lipid accumulation and plaque formation within arterial walls^1^, is the primary condition underlying cardiovascular disease (CVD), the leading cause of global mortality and morbidity^2,3^. Despite significant advancements in pharmacotherapies for lipid lowering and the management of other vascular risk factors such as diabetes and hypertension, the alarmingly high and rising prevalence of CVD highlight the need for novel risk assessment and preventive strategies^4,5^. Atherosclerosis is a chronic disease with subclinical atherosclerotic lesions developing silently over decades. Current CVD risk assessment tools used in clinical practice, including the Pooled Cohort Equations (PCE)^6^, Framingham Risk Score^7^, and Systematic Coronary risk Evaluation (SCORE^8^ and SCORE2)^9,10^, rely on demographic, clinical, and biochemical factors but do not account for the presence of subclinical atherosclerosis^11,12^. Imaging studies that enable screening for subclinical atherosclerotic lesions in asymptomatic individuals suggest that atherosclerotic plaques are highly prevalent, even among individuals traditionally considered at low CVD risk^13–18^. Coronary artery calcium (CAC) scoring on computed tomography (CT) has gained traction for assessing subclinical atherosclerosis; however, it faces limitations as a screening tool due to ionizing radiation exposure and its relatively high costs for widespread application^19,20^.

In contrast, carotid ultrasound offers a non-invasive, radiation-free, and widely accessible modality for assessing subclinical atherosclerotic lesions^19,21^. While traditional assessment of carotid intima-media thickness (cIMT) does not reliably predict incident CVD risk^22^, the detection of carotid atherosclerotic plaques is associated with an increased risk of future events^21,23,24^. Despite promising results, it remains uncertain whether screening for carotid plaques could reclassify asymptomatic individuals into higher-risk categories that justify the initiation of preventive pharmacotherapies^25^. Many published studies are constrained by relatively small sample sizes or insufficient follow-up^24,26–30^.

Several large-scale population-based cohorts have incorporated carotid ultrasound imaging into their data collection processes,^31–33^ but evaluating plaque presence across thousands of images remains a labor-intensive task. Recent advancements in deep learning have enhanced medical imaging analysis, enabling greater precision and the automation of processing large volumes of imaging data^34,35^. Although previous deep learning models for ultrasound images have shown potential in various tasks, including carotid wall and plaque segmentation^36–38^, these studies have primarily focused on specific populations, such as stroke patients or individuals with known carotid artery disease, which limits the generalizability of their findings to the broader population. Automating carotid plaque assessment in large population-based cohorts could allow the integration of this phenotype with genetic, omics, other imaging modalities, and clinical data collected in the context of these studies. This would facilitate in-depth research into the biology of subclinical atherosclerosis, thus enabling explorations into the natural history of the disease and potentially uncovering novel drug targets.

Here, we introduce a computer vision model designed to detect atherosclerotic plaques, applied to the largest dataset of carotid artery ultrasound images to date. We utilized ultrasound images from 19,499 deeply phenotyped participants in the UK Biobank (UKB), a large-scale population-based cohort. This study marks the first application of deep learning at a population level for assessing subclinical atherosclerosis using carotid ultrasound, offering a valuable resource for exploring the biology of atherosclerosis with implications for CVD risk assessment. Our model demonstrated high performance in both detecting plaques and quantifying their counts. We leveraged the model’s predictions to: (1) estimate the prevalence of carotid atherosclerotic lesions; (2) identify predictors of carotid plaque presence; (3) examine the associations of plaque presence and count with the risk of future CVD events; (4) assess potential improvements in CVD risk prediction and reclassification compared to traditional clinical tools; and (5) investigate the genetic underpinnings of carotid atherosclerosis (Figure 1).

## Results

### Study population

A total of 177,757 images from 19,499 participants who underwent carotid ultrasound during the first imaging visit of the UKB, were available for analysis (Supplementary Figure 1). The protocol for carotid artery examination has been described previously^39^. For the current study, we used 38,732 images obtained in the longitudinal axis of the left and right distal common carotid artery and the bifurcation, allowing for the assessment of plaque presence along the vessel wall. The demographic and medical characteristics of the study participants are presented in Table 1. The mean age at the time of the carotid ultrasound examination was 64.6 years (SD = 7.59), and 50.8% of the participants were female. A total of 1,381 (7.1%) study participants had a baseline diagnosis of atherosclerotic CVD. A comparison between UKB participants with carotid ultrasound and the rest of the UKB cohort revealed a lower prevalence of CVD risk factors (Supplementary Table 1).

### Plaque detection model

**Figure 2.**
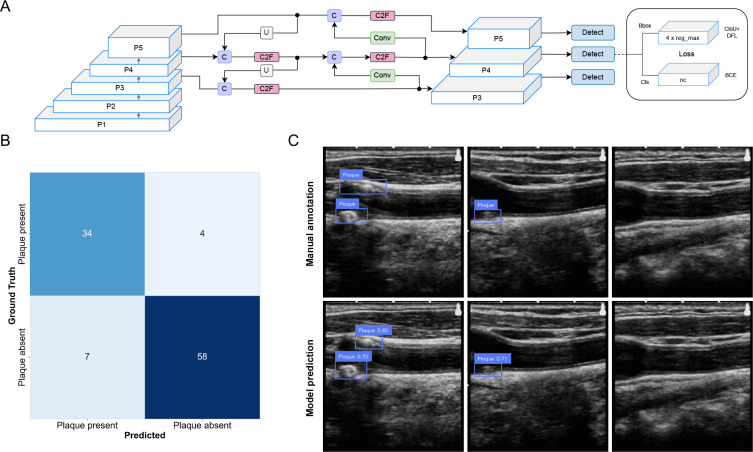
Development and performance of the plaque detection model. **A**. General representation of the YoloV8 architecture, C – concatenation, C2F – cross-stage partial bottleneck with two convolutions, U – up-sampling, Conv – convolutional module, P1-P5 represent future maps; Bbox – bounding box prediction branch, Cls – classification branch, BCE – Binary Cross Entropy loss, DFL – Distribution Focal Loss, CIoU – Complete Intersection over Union, nc – number of classes, reg_max – maximum value for the bounding box regression. Adopted from Terven & Cordova-Esparza^42^ and https://github.com/ultralytics/ultralytics/issues/189. **B**. Confusion matrix for model’s classification performance for plaque presence at each image. The confusion matrix is based on comparing the presence of plaque in the annotations with the model’s predictions. Specifically, if there is a plaque annotation for an image and the prediction contains a bounding box, then the prediction is annotated as true positive. **C.** Examples of model predictions as compared to manual annotations for three UK Biobank participants in the test set. Each image depicts the longitudinal view of the common carotid artery extending toward the bifurcation area (the left part of the images).

To train a deep learning model for the detection of atherosclerotic plaques, we manually annotated plaques in 680 randomly selected carotid ultrasound images. Plaques were defined as focal protrusions in the arterial lumen with a thickness greater than 50% of the surrounding carotid intima-media thickness^40^. A plaque was present in 253 of these images. We performed transfer learning with fine-tuning by employing a pre-trained YOLOv8^41^ object detector as the foundation for developing the plaque detection model (Figure 2A). The YOLOv8 object detection algorithm generates bounding boxes to indicate the locations of objects of interest. The images with manually annotated plaques were randomly divided into training, validation, and test sets in a 0.725/0.125/0.15 ratio. This distribution maximized the training set while ensuring a sufficient number of images for assessing model performance in the test set. We evaluated the model’s performance by training it on several subsets of our input development dataset (training + validation) in 5-fold cross-validation. The consistent performance metrics, with minimal variations in precision and recall, indicated no significant signs of overfitting (Supplementary Figure 2).

After training, the performance of the model was evaluated in a blind test set of 103 images, of which 38 contained at least one plaque (53 plaques in total). The model achieved high classification metrics for plaque presence at the image level, with an accuracy, sensitivity, specificity, and Positive Predictive Value (PPV) of 89.3%, 89.5%, 89.2%, and 82.9% (Figure 2B), respectively, at an iteratively tuned confidence score threshold of 13%. The confidence score measures the model’s certainty that a box contains an object of interest and was tuned to optimize and balance accuracy, sensitivity, and specificity. The Mean Average Precision at an Intersection over Union (IoU) threshold of 50% (mAP@50) was 68.4%, indicating the precision with which the model can localize objects with at least 50% overlap with the ground truth. The model’s detection precision and recall were 70.3% and 71.7%, respectively. Prediction examples are illustrated in Figure 2C and Supplementary Figure 3.

### Prevalence and risk factors of carotid plaques in the UK Biobank

Next, we deployed the model on all available long-axis carotid ultrasound images from the UKB cohort (38,732 images, 19,499 individuals, Supplementary Figure 1). This deployment allowed us to extract data on plaque phenotypes, including plaque presence and the count of plaques in either artery for each individual. The count of plaques was determined by the number of predicted bounding boxes in each image.

**Figure 3.**
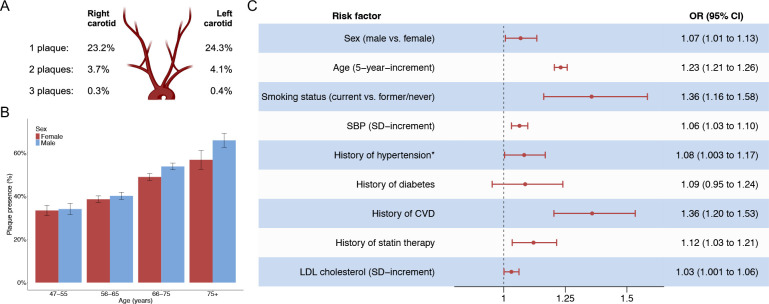
Prevalence and predictors of carotid plaque in the UK Biobank. **A**. Distribution of plaques in the left and right carotid arteries. **B**. Percentage of plaque presence across age and sex groups. The error bars represent 95% confidence intervals. **С.** Forest plot of the associations of demographic and vascular risk factors with the odds of carotid plaque presence, as derived by a multivariable logistic regression model that includes all variables in the figure. The results are presented as odds ratios (OR) and 95% confidence intervals (CI). SBP – systolic blood pressure; CVD – cardiovascular disease; LDL – low-density lipoprotein cholesterol; SD – standard deviation. * History of hypertension is defined by the use of antihypertensive medication

Overall, the model detected at least one plaque in 45% of the UKB participants who underwent a carotid ultrasound examination. In 14% of the participants, the model detected at least two plaques, and in 3.1% of the participants, three or more plaques across both arteries. The prevalence of plaques in the left and right carotid arteries is presented in Figure 3A. As a quality control step for the model predictions, we explored whether plaque presence was associated with cIMT, which was quantified and documented for each individual at the time of the imaging assessment. Indeed, cIMT was consistently higher for individuals for whom our model predicted a plaque (Wilcoxon test p < 10^−60^ for maximum, mean, and mean of maximum cIMT measurements). Similar results were obtained for the left and right arteries separately (Supplementary Figure 4).

Plaques were more common in male participants (47.5% vs. 42.6%; two-proportions z-test p = 5.7 × 10^−12^) and plaque prevalence was significantly associated with older age, increasing from 31.1% in participants aged 45–54 years to 62% in participants aged 75 years or older (Cochran-Armitage trend test p = 1.12 × 10^−88^, Figure 3B). In a multivariable logistic regression model, male sex, older age, current smoking, higher systolic blood pressure (SBP), history of hypertension, pre-existing CVD, use of statins, and higher Low-Density Lipoprotein (LDL) cholesterol levels were all significantly associated with plaque presence (Figure 3C).

### Associations of plaque phenotypes with the risk of future cardiovascular events

To estimate the effects of the presence and count of plaques in carotid ultrasound on the risk of future major adverse cardiovascular events (MACE), we conducted a survival analysis. Following the first imaging visit, the UKB participants with available ultrasound images were followed up for a median of 55 months (range 1–80 months). During this time interval, a total of 430 individuals experienced a MACE, defined as myocardial infarction, stroke or death due to any cardiovascular cause. Of these, 335 were first-ever events among 18,110 participants (1.8%) without a history of CVD at the time of the ultrasound examination, while 95 were secondary events among 1,389 participants (6.8%) with an existing history of CVD.

**Figure 4.**
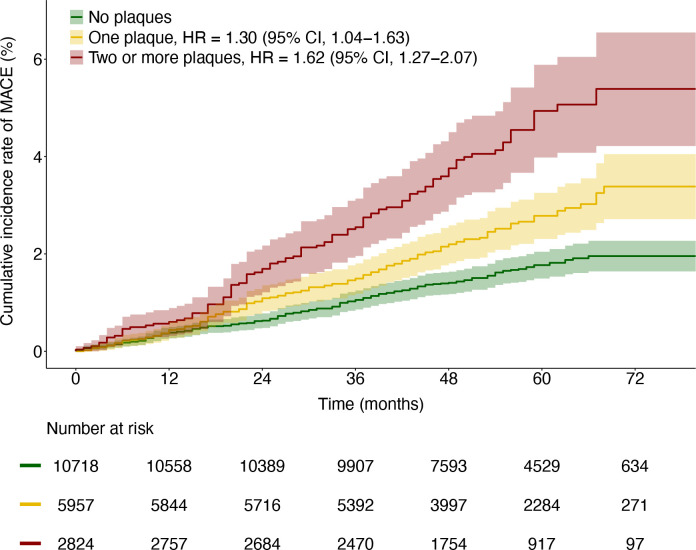
Survival curves of cumulative major adverse cardiovascular event (MACE) rates by total count of carotid plaques predicted by the model. The presented hazard ratios (HRs) were estimated using Cox regression, adjusted for: sex, age, systolic blood pressure, use of statins, history of antihypertensive therapy, current smoking, history of diabetes, HDL cholesterol and total cholesterol levels.

Kaplan-Meier estimates indicated a higher incidence rate of MACE among individuals with carotid plaques compared to those without plaques, demonstrating a dose-response pattern of higher incidence with an increasing plaque count (log-rank test p-value for all pairwise comparisons < 0.05, Figure 4). After adjusting for conventional cardiovascular risk factors in Cox regression models, plaque presence was significantly associated with future risk of MACE (Hazard Ratio (HR) 1.42, 95% CI: 1.16–1.73). The plaque count per individual showed a dose-dependent association with future CVD risk (HR for 1 plaque vs. no plaque = 1.30, 95% CI: 1.04–1.63; HR for 2 or more plaques vs. no plaques = 1.62, 95% CI: 1.27–2.07). These associations were consistent in the subgroup of individuals without an existing history of CVD, as well as in those who had neither a history of CVD nor statin use (Supplementary Figure 5). There was no evidence of an interaction with sex (p = 0.139). The HRs were comparable when analyzing individual MACE components, albeit with wider 95% CIs, probably due to lower statistical power (195 myocardial infarction and 172 stroke cases, Supplementary Figure 5).

### Predictive power of plaque phenotypes

To examine whether assessing carotid plaque phenotypes could improve cardiovascular risk prediction, we compared the fitness, reclassification, and discrimination of prediction models that included conventional vascular risk factors with those that also considered plaque presence and count. Both plaque presence and count significantly improved the overall goodness of fit of a Cox regression model (P < 0.05), as assessed by the log-likelihood ratio test^43^. We observed a reclassification improvement for plaque presence (category-free net reclassification improvement (cfNRI): 0.331, 95% CI, 0.217–0.445) and plaque count (cfNRI: 0.369, 95% CI, 0.260–0.478, Table 2). Briefly, NRI quantifies model improvement by the difference between the net proportion of cases for which the new model correctly increases predicted risks and net proportion of controls for which the new model correctly decreases predicted risks^44,45^. Sensitivity analyses confirmed these improvements in individuals without a history of CVD and statin use (Table 2). Despite the strong associations with the future risk of MACE, none of the plaque phenotypes added to conventional risk factors significantly improved model discrimination, as measured by the C-index. Specifically, adding plaque presence to conventional risk factors only slightly changed C-index from 0.745 (95% CI, 0.723–0.767) to 0.747 (95% CI, 0.725–0.769), while adding plaque count changed it to 0.748 (95% CI, 0.726–0.770). However, minor yet significant improvements were observed in the integrated discrimination improvement (IDI): 0.0022 (95%CI, 0.0002–0.0050) for plaque presence and 0.0023 (95%CI, 2×10^−5^–0.0063) for plaque count. All the models demonstrated good calibration, indicating a strong alignment between observed outcomes and predicted risk estimates (Supplementary Figure 6).

To assess whether plaque phenotyping would improve the reclassification of individuals when complementing established clinical risk prediction models, we calculated the PCE risk scores for UKB participants eligible for assessment according to ACC/AHA guidelines^6^. Due to the PCE’s tendency to overestimate risk in the UKB population, the model was recalibrated (Supplementary Figure 7). Incorporating plaque presence and count into the PCE demonstrated significant reclassification improvement, with a categorical NRI of 0.034 (95% CI, 0.006–0.062) and 0.04 (95% CI, 0.010–0.070), respectively, at the threshold of 7.5% 10-year cardiovascular risk, which defines intermediate risk and justifies preventive initiation of statin therapy according to current guidelines. Overall, adding plaque presence correctly reclassified 17 out of 318 patients who developed MACE into a higher risk category. Including plaque count correctly reclassified 20 patients into a higher risk category (Table 3). In both cases, plaque presence and plaque count,4 patients were incorrectly reclassified into a lower risk category.

### Genome-wide association study of carotid atherosclerotic plaque

**Figure 5.**
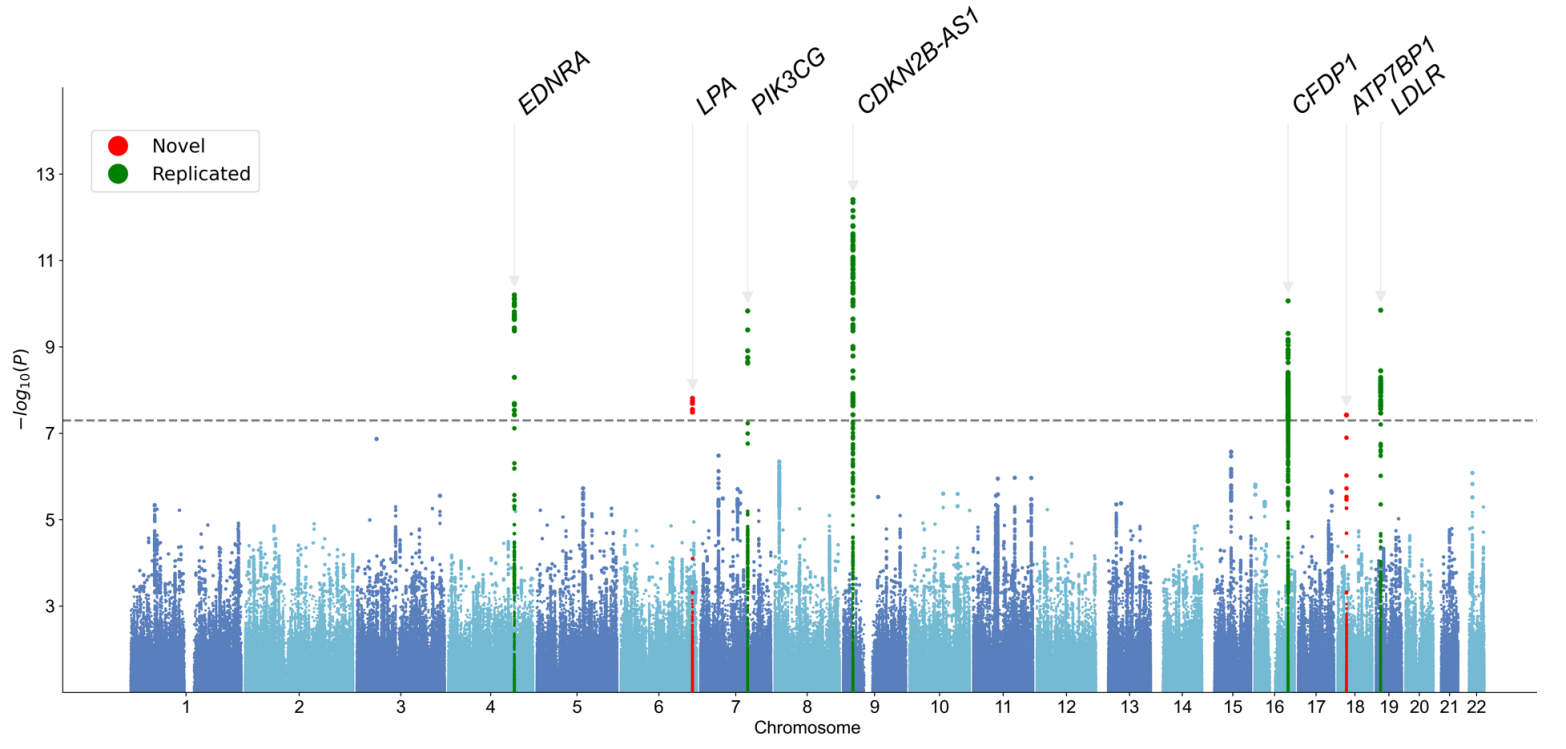
Manhattan plot of the GWAS meta-analysis for carotid plaque presence (29,790 cases; 36,847 controls). Loci highlighted in red point to novel significant associations for carotid plaque whereas loci highlighted in green represent validation of previously described associations.

As the next step, we investigated the genetic architecture of carotid atherosclerosis, defined by plaque presence. To detect single nucleotide variants (SNVs) associated with presence of a carotid atherosclerotic plaque, we conducted a genome-wide association study (GWAS) and subsequently meta-analyzed our data with the largest available GWAS for carotid plaque from the cohorts of the Cohorts for Heart and Aging Research in Genomic Epidemiology (CHARGE) consortium^46^. After quality control and excluding individuals without genetic data, the UKB GWAS included 18,203 White British individuals, comprising 8,250 cases with carotid plaque and 9,953 controls. The pooled sample from the UKB and CHARGE cohorts included 66,637 individuals (29,790 cases; 36,847 controls). We identified seven independent genomic loci significantly associated with the presence of carotid plaque, two of which were novel. Five of the loci (mapped to the genes *EDNRA, LINC02577, CDKN2B-AS1, CFDP1, LDLR*) replicated known associations, as the lead SNVs were in high linkage disequilibrium (r^2^ > 0.9) with SNVs previously reported to be associated with carotid plaque presence in the CHARGE study (Figure 5, Supplementary Table 2). The sixth locus included the *LPA* gene, which encodes lipoprotein(a) (Lp(a)) and is a known locus for atherosclerotic cardiovascular disease^47–51^. The lead variant at this locus (rs56393506), associated with higher odds for an atherosclerotic plaque (OR for T allele: 1.12, 95%CI: 1.07–1.16), is an intronic variant in the *LPA* gene that has been previously strongly associated with higher Lp(a) levels^52^. The lead SNV at the seventh locus is located in a non-coding region (rs1893250, OR for A allele: 0.91, 95%CI: 0.88–0.93) and was previously associated with angina pectoris^53^.

### Mendelian randomization analyses

**Figure 6.**
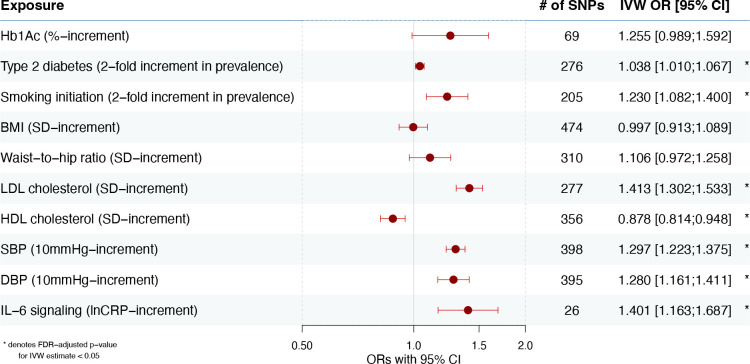
Forest plot for Mendelian randomization results. The results are presented as odds ratios (OR) and 95% confidence intervals (CI) derived from random-effects inverse-variance weighted Mendelian randomization analyses. Two-fold increments in prevalence for binary exposures (type 2 diabetes and smoking initiation) were derived by multiplying the IVW betas and corresponding confidence intervals by 0.693, as described by Burgess and Labrecque^54^. HbA1c – Glycated hemoglobin; BMI – Body Mass Index; LDL – Low-Density Lipoprotein Cholesterol; HDL – High-Density Lipoprotein Cholesterol; SBP – Systolic Blood Pressure; DBP – Diastolic Blood Pressure; IL-6 – Interleukin-6.

Finally, we performed Mendelian Randomization (MR) to explore whether genetically proxied risk factors and biomarkers of CVD are associated with carotid atherosclerotic plaque. We used the largest to-date publicly available GWAS summary statistics for vascular risk factors to generate genetic instruments for the risk variables under study (Supplementary Table 3). The inverse-variance weighted (IVW) MR analyses revealed associations between higher genetically proxied SBP, diastolic blood pressure (DBP), LDL cholesterol, interleukin-6 (IL-6) signaling activity, and genetic predisposition to smoking initiation and type 2 diabetes (T2D) with the odds of carotid plaque presence (Figure 6). Additionally, higher genetically proxied HDL cholesterol levels were associated with lower odds of carotid plaque (Figure 6). There was evidence of directional pleiotropy, as assessed by a significant Egger intercept (p = 0.036), for the association between smoking initiation and carotid plaque, with the MR estimate derived by MR Egger regression being in the opposite direction, even after excluding potential outlier instruments detected with Mendelian Randomization Pleiotropy RESidual Sum and Outlier (MR-PRESSO) (Supplementary Table 4). The results for the remaining significant IVW associations were generally highly consistent in sensitivity analyses, including MR Egger regression and the weighted median estimator (Supplementary Table 4).

## Discussion

In this study, we developed a computer vision model that accurately detects atherosclerotic plaques in carotid ultrasound images and applied it to a population-based cohort of 19,499 participants from the UKB. The model demonstrated strong performance in detecting carotid plaques and classifying plaque-positive carotid ultrasound images, achieving approximately 90% in accuracy, sensitivity, and specificity. Consistent with previous studies in comparable demographics (mean age 64.6±7.6 years, 53% female)^14,55^, our model identified at least one carotid plaque in 45% of participants. The presence of plaques was associated with conventional vascular risk factors and was predictive of future adverse cardiovascular events over a follow-up period of up to 7 years. Importantly, both plaque presence and count led to improved risk reclassification for future adverse cardiovascular events beyond the established PCE risk assessment tool. Leveraging the phenotypic depth of the UKB, we conducted the largest genomic analysis of carotid atherosclerosis to date, identifying two novel loci and risk pathways, including ones targeted by emerging cardiovascular therapeutics, such as Lp(a) and IL-6 signaling.

While atherosclerosis can develop long before clinical symptoms appear^56^, modern risk assessment tools do not consider subclinical atherosclerotic pathology. Many studies have highlighted the potential of integrating imaging biomarkers of subclinical atherosclerosis into conventional risk assessment tools^28^. CAC assessed through CT is a well-established predictor of CVD risk^57^. However, CAC has limitations, including insensitivity to early stages of atherosclerosis and exposure to radiation^19,20,58^. In contrast, carotid ultrasound is an inexpensive, well-tolerated, radiation-free tool capable of detecting early-stage atherosclerosis^14,59^. Our results, based on a population of almost 20,000 individuals, suggest that both the presence and count of plaques are strongly associated with the risk of future acute CVD events. Incorporating plaque information into Cox regression models led to significant reclassification improvements, which were robust across the full cohort, those without a history of CVD statin-naive participants. The reclassification metrics in our study indicate that adding plaque information to conventional risk factors and directly incorporating it into the PCE has the potential to improve patient stratification. Specifically, incorporating plaque count reassigned 6.3% (20 out of 318) of individuals who went on to develop cardiovascular events from a low to a higher risk category, making them eligible for preventative statin therapy.

Despite growing evidence that carotid ultrasound-derived plaque phenotypes—such as total plaque area and vulnerability features—are independently associated with future CVD events^26,60,61^, its use in clinical practice remains underutilized. The primary challenges for wider adoptions include the time required for assessment and reliance on operator skill^23^. However, an efficient artificial intelligence model could significantly streamline these labor-intensive tasks. The model developed in this study demonstrates high performance in identifying individuals with carotid atherosclerosis and localizing plaques from a single screenshot of the carotid bifurcation. Further model advancements could not only address the issue of labor intensity but also enable the extraction of more detailed plaque features, thus enhancing the predictive performance of carotid ultrasound.

Importantly, our model enhances the phenotypic depth of the UKB to subclinical atherosclerosis, facilitating integration with the unique resources available in this population, such as multi-omics and other imaging data^62^. Moving in this direction, we leveraged the available genomic data to perform the largest exploration to date of the genetic architecture of carotid plaque. We replicated five genomic loci previously associated with atherosclerosis endophenotypes^46^ and also found two new loci related to clinical cardiovascular outcomes. Furthermore, downstream MR confirmed the effect of genetic predisposition to known vascular risk factors, such as smoking, high blood pressure, and LDL cholesterol, on carotid plaque presence. Importantly, the GWAS and MR results showed that genetic variation leading to elevated Lp(a) levels and higher IL-6 signaling activity is associated with higher odds of carotid plaque. Both pathways are believed to play key roles in atheroprogression and are the targets of investigational drugs in advanced clinical development^47,48,63–65^. These results suggest that drugs targeting these pathways could be promising, particularly in the preclinical stages of atherosclerosis. Integrating carotid plaque phenotypes with additional omics layers may provide further insights into novel drug targets for atherosclerotic CVD.

Our study has several limitations. First, during model development, the low quality of some images necessitated contrast enhancement and noise reduction techniques. These adjustments may have introduced bias, especially in cases where high noise levels complicated plaque detection. However, our model achieved high classification and detection metrics, which could potentially be improved further by annotating more images. Moreover, the consistency of predicted carotid plaque prevalence with previously reported plaque prevalence in similar demographics, along with the associations of predicted plaque presence with known risk factors and future CVD risk, supports the model’s reliability^55^. Second, the UKB is a cohort of healthy volunteers with a lower incidence rate of CVD than the general population, particularly within its imaging subsample (Supplementary Table 1)^66^. This discrepancy contributes to an overestimation of CVD risk calculated by clinical risk models, such as the PCE tool. To improve the reliability of our reclassification evaluation, we recalibrated the model using data from the study population to better align predicted risk with observed outcomes. Due to the low prevalence of risk factors and events, PCE-derived absolute risk estimates remain notably low. It is worth noting that deriving categorical NRI metrics comparable to those from other studies with different incidence rates may be problematic^67^. We addressed this issue by calculating continuous NRI estimates using bootstrap estimates, which are threshold-independent and less sensitive to event rates^68^. Third, the carotid ultrasound examination took place 2 to 15 years after the baseline visit and the assessment of cardiovascular risk factors. This gap introduces bias into the effect estimates. To account for changes in baseline risk factors over this period, we used, wherever available, data from the primary care records of participants collected at the closest date to the ultrasound exam. Fourth, our study has a shorter follow-up period of 7 years compared to most clinical risk assessment tools, including the PCE, which typically calculate 10-year risk estimates for CVD. Fifth, we observed significant heterogeneity in the results of IVW MR analyses for several risk factors (Supplementary Table 4), which could indicate the presence of pleiotropy. To explore whether the derived estimates could be biased by directional pleiotropy, we conducted several sensitivity analyses to test the robustness of the estimates against different MR assumptions. Sixth, there was some population overlap between the exposure and outcome GWAS datasets used in our MR analyses, which could introduce weak instrument bias into the derived effect estimates. We addressed this concern by using the largest available summary statistics for the exposure data. Given that the effective population overlap was less than 5% for all exposure-outcome pairs, we estimated any bias in the effect estimates to be under 5%^69^. Lastly, this study analyzed a sample from the UKB, which predominantly consists of White ancestry volunteers, who are healthier than the general population. Therefore, replicating these findings in more diverse populations and real-world settings is crucial for improved generalizability and informed decision-making.

In conclusion, we have successfully developed and implemented a deep learning model for plaque detection within the population-based UKB, significantly enhancing the phenotypic characterization of this cohort. This model sets the stage for automating carotid plaque assessment in other large-scale cohorts, thereby enabling broader population-based research in subclinical atherosclerosis. Our results highlight the potential of carotid plaque assessment for refining cardiovascular risk prediction, offer insights into the genetic architecture of atherosclerosis, and provide a valuable resource for advancing atherosclerosis research at the population scale.

## Methods

### Study population

In this study, we utilized data from the UKB, a large-scale prospective cohort study that recruited between 2006 and 2010 502,422 individuals aged 40 to 69 at baseline from across the United Kingdom^70^. Participants underwent detailed assessments, which included comprehensive data collection through questionnaires, physical measurements, and biological sample collections. All participants provided electronic informed consent. Ethical approval was granted by the National Health Service North West Multicenter Research Ethics Committee.

Following a baseline visit between 2006 and 2010, a total of 82,340 individuals returned for a follow-up imaging visit starting 2014, which included a carotid ultrasound. A total of 177,757 raw images from 19,768 individuals were released by the UKB and used in this study (Supplementary Figure 1). Four anatomic views of the distal common carotid artery and the bifurcation were available for each side for nearly every UKB participant who underwent a carotid ultrasound: images along the main longitudinal axis, images along the short axis, and images along the main longitudinal axis at two different angles for each artery, which were used by the analysts for cIMT quantification. Our study focused on images from 19,507 UKB participants derived along the main longitudinal axis. Eight individuals withdrew from the study post-recruitment (field 190), resulting in a total sample size of 19,499. For participants with repeat imaging visits, only the ultrasound data from the first visit were retained for analysis.

### Pre-processing

The flowchart for extracting the carotid ultrasound imaging data for analysis is summarized in Supplementary Figure 1. After developing an algorithm (Supplementary Figure 8) that automatically detects images along the longitudinal axis, we extracted 45,210 long-axis images without cIMT measurements from the left and right arteries for 19,507 individuals. The obtained images were cropped to a size of 480×448 pixels to retain only the ultrasound image while maintaining the original resolution. After excluding participants who withdrew from the study and keeping only the images from the first ultrasound visit, a total of 19,362 left and 19,370 right carotid images from 19,499 individuals remained for analysis.

In order to enhance contrast and reduce noise in the images, we applied two functions from the *OpenCV* v. 4.7.0 library^71^: median blur filtering (ksize=5) and Contrast Limited Adaptive Histogram Equalization (clipLimit=2.0, tileGridSize=(8,8)), respectively, to facilitate the manual segmentation process. These processing steps were applied to the full sample of images in this study. Plaques were manually annotated by two medical doctors with postgraduate training in vascular imaging and subsequently validated by a doctor certified in carotid ultrasound imaging. *Label Studio* version 1.8.2 (https://github.com/HumanSignal/label-studio) was used to segment plaques on the ultrasound images. The edge coordinates of the segmentation masks were used to obtain the bounding boxes. Plaques were defined according to standards, as focal protrusions in the arterial lumen with a thickness >50% of the surrounding carotid intima-media thickness^40^. If multiple longitudinal images were available for the same artery, those where the model detected a plaque were prioritized, or a random image was used if plaques were found in more than one.

### Model Development and Deployment

We performed transfer learning with fine-tuning which involves selecting a model pre-trained on a large dataset of natural images and then re-training it on a new dataset. This approach allows for adjusting the model weights and biases to better suit the task related to the new dataset. Here, we employed the YOLOv8l^41^ model for object detection, pre-trained on over 330,000 images, and re-trained it on our dataset of 680 carotid ultrasound images. The dataset was divided into training, validation, and test sets at a ratio of 0.725/0.125/0.150, resulting in 103 images allocated to the test set. To enhance model generalizability and predictive power, we randomly selected 50% of the training set (490 images) and applied various augmentation techniques from *Albumentation*s^72^ Python library: GridDistortion (p=0.15), RandomBrightnessContrast (((0,0.5),(0,0.5)), HorizontalFlip(p=0.2), GaussNoise(p=0.15), and RandomSizedCrop (min_max_height=(384, 384), p=0.4). These augmentations increased the variability of the training set, making the model more invariant to noise and other distortions. This augmented dataset, along with the rest of the images, was processed in batches for further augmentation within the YoloV8 framework, as detailed below.

The model was trained with a batch size of 44 images. Early stopping was set to 5 epochs, and training concluded after 14 epochs, with peak performance observed at epoch 9. The following loss function parameters were selected based on a grid search, as detailed in Supplementary Table 5: distribution focal loss (DFL) = 2.5, box loss = 10, and binary cross entropy loss (CLS) = 1.1. Default augmentation techniques in the YOLOv8 framework were partially suppressed due to prior augmentation efforts; specifically, mosaic, copy-paste, shear, close mosaic, flip up-down, and mix-up were disabled. However, flip left-right (p=0.1), degrees (10), HSV-Saturation (hsv_s: 0.05), HSV-Value (hsv_v: 0.05), translate (0.1), and scale (0.1) were retained. Training was conducted using an NVIDIA QUADRO RTX 5000 GPU (16GB). *PyTorch*^73^ version *1.12.1* and the *Ultralytics*^41^ framework version *8.1.16* were used for model development.

Model’s classification metrics for detecting plaque-positive images were estimated in a confusion matrix by comparing ground truth annotations with bounding box predictions. A true positive was recorded when both annotation and prediction contained a bounding box.

### Demographic and Clinical Variables

Age at carotid ultrasound assessment was derived by subtracting the participant’s date of birth (field 33) from the carotid ultrasound assessment visit date (extracted from the manifest files linked to the ultrasound images). Ethnicity for the PCE risks score calculation was determined by field 21000. Observations with missing data or responses of “Do not know” or “Prefer not to answer” were encoded as “Other”. Pre-existing CVD was defined based on self-reported history (UKB field 20002, with 1075 - heart attack/myocardial infarction, 1081 - stroke, 1583 - ischemic stroke, 1491 - brain haemorrhage); general practice records (131298, 131300, 131302, 131368, 131366); hospital data records (defined using ICD-10 codes I20-I25, I60-I61, and ICD-9 codes 410–412, 429–431, 434, 436, as well as operation codes K40–46, K49, K471, K49, K50, K75, L294 and L295 observed before the initial ultrasound visit). SBP was quantified using fields 4080 and 93 by averaging the observations from each field, followed by taking the mean of the resulting values. Smoking status was categorized as “current” or “other”, with missing data (<0.5%) treated as “other” (field 20116). Total cholesterol, LDL and HDL cholesterol levels were obtained from fields 30690, 30780 and 30760, respectively. When available, values from the assessment closest to the ultrasound visit were extracted; otherwise, baseline measurements were used. Missing values were imputed using the multivariate imputation by chained equations method^74^, affecting 12% of HDL-cholesterol values and 6% of total and LDL-cholesterol values, based on other CVD risk factors. Diabetes was defined by self-reported cases (UKB field 20002 – codes: 1220, 1222, 1223), use of glucose-lowering medications (field 20003), and hospital records prior to the first carotid ultrasound exam (ICD-9 codes: 250* and ICD-10 codes E10, E11). Information on the use of antihypertensive drugs, diabetes medications, and statins was obtained from field 20003 (Supplementary Table 6).

### Assessment of major adverse cardiovascular events

MACE endpoints were defined as follows: myocardial infarction (ICD-10 codes I21, I22 from hospital inpatient data, UKB data-fields 131298 and 131300), stroke (ICD-10 codes I60–64 from hospital inpatient data, UKB data-fields 131368 and 131366), or death due to any cardiovascular cause (defined as the cause of death with an ICD-10 code starting with ‘I’ extracted from the death registry). The date of the first episode observed after the carotid ultrasound assessment was considered as the date of the event of interest.

### Carotid intima media thickness

We calculated three types of cIMT measurement characteristics between groups with and without carotid plaque, as predicted by the model. Measurements of mean and maximum cIMT were obtained using UKB data fields 22670–22681, as described by Strawbridge et al^75^.

- cIMT mean: average of the mean values from four mean cIMT measurements (two angles for each carotid artery: left and right).
- cIMT max: The maximum value of the cIMT measurements across both arteries.
- cIMT mean-max: The mean of the maximum cIMT values per artery.

All the obtained values were log-transformed for the analysis. Individuals with more than one missing cIMT measurement were excluded, resulting in a total of 18,497 individuals for this analysis. The analysis was repeated separately for the right and left arteries.

### Logistic regression for plaque presence and Cox regression for future events

To estimate the associations between model-derived plaque presence and CVD risk factors, we applied logistic regression, using plaque presence as the outcome variable. A history of hypertension was defined by the use of antihypertensive treatment at the time of the carotid ultrasound assessment. Definitions of other clinical and demographic variables are described above.

For each subset of the cohort (full sample, primary events with and without individuals on statin therapy), two separate Cox regression models were constructed: one with plaque presence as a binary variable and another with the count of plaques as a categorical variable with three levels (no plaques as the reference, one plaque, and two or more plaques). The time variable in the survival analysis was calculated as the duration from the first ultrasound visit to the event of interest or to the censoring date, which included date at death from causes other than CVD or the date at the last observed event (2022-10-30) available at the time of the analysis. Controls were censored at the time point of the latest observed event. The fitted Cox regression models included all vascular risk factors included in the PCE (age, sex, SBP, smoking status, history of diabetes, antihypertensive therapy, cholesterol, and HDL cholesterol), as well as statin usage. Ancestry was not included due to its very low variance in our sample (Table 1). The proportional hazard assumptions were tested with the scaled Schoenfeld residuals, and no violation of the assumptions was detected.

All analyses were conducted using R software, version 4.4.0. We considered two-sided p-values less than 0.05 to be statistically significant. The category-free NRI was calculated using the *nricens* v.1.6^76^ package with confidence intervals and p-values based on 1000-fold bootstrap standard errors. The IDI metric was calculated using the *survIDINRI* library v.1.1–2^77^. Harrell’s C-statistic, along with its 95% confidence interval, was used to evaluate the discriminative ability of the time-to-event models^78^. Comparisons between models based on the C-statistic were conducted using the *CsChange* package^79^.

### PCE risk estimation

The calculation of the PCE risk score was performed using published equations^6^. PCE eligibility included the following criteria:

1. 40≤Age≤79,
2. 130 ≤ Total cholesterol ≤320 mg/dL;
3. 20 ≤ HDL-cholesterol ≤ 100;
4. 90 ≤ Systolic blood pressure ≤ 200 mmHg

To enable fair model comparison and justify the recommended threshold usage, the original PCE was calibrated to the UKB population. This recalibration was achieved by fitting calculated log-hazards from the published PCE coefficients in a Cox regression stratified by sex to obtain recalibrated probabilities. The calibration of the predicted risk values from the original and recalibrated models was assessed using Greenwood-Nam-D’Agostino statistics and the integrated calibration index^80,81^. Calibration plots for the PCE are presented in Supplementary Figure 7.

The plaque variables were incorporated into the PCE, with plaque presence as a binary variable and plaque count as an ordinal variable, as previously described^82^. Briefly, the recalculated risk is derived from the relative risk estimate for the novel risk factor, the baseline risk, and the prevalence of the novel risk factor. As an example, the presence of plaque was incorporated using equation (1), adapted from Kooter et al^82^:

(1) $$[r]=p\times HR+(1-p)\;MF_{\left(+\right)}=HR/[r]\;R_{\left(+\right)}=R_{bl}/[r]\;R_{\left(-\right)}=R_{+}/RR$$

where [r] represents the weighted mean risk; p is the plaque prevalence; HR is the hazard ratio for plaque presence; ${MF}_{\left(+\right)}$ is the multiplication factor; $R_{\left(+\right)}$ is recalculated risk in the presence of plaque; $R_{bl\;}$ is the baseline risk estimated from the baseline hazard and log-HRs estimated from the fitted Cox model; and $R_{\left(-\right)}$ is the recalculated risk in the absence of plaque.

HRs for both plaque presence and plaque count, used to enhance the recalibrated PCE risk model with plaque information, were estimated from a Cox regression model fitted on the sub-cohort eligible for PCE risk assessment. The model was adjusted for sex, age, HDL and total cholesterol levels, antihypertensive drug use, smoking status, SBP, and statin use at the time of the initial ultrasound assessment. Plaque information was incorporated separately for males and females, with prevalence estimates calculated for each cohort.

Model calibration was estimated using the survival.calib^83^ package in R. Reclassification tables and NRI statistics were calculated using the *PredictABEL* v.1.2–4^84^ library in R.

### Genome-wide association study and meta-analysis

Genomic quality control in the UKB was performed as previously described^85^. For the GWAS analysis *Regenie*^86^ v3.3 was employed. In step 1, we used directly genotyped variants with MAF > 1%, <10% missingness, Hardy–Weinberg equilibrium test *P* > 1 × 10^−15^, and a minor allele count > 100. Age at the time of the ultrasound exam, along with sex, the first 10 genetic principal components, and the genotyping chip were used as covariates. Fixed-effect meta-analysis was conducted with *METAL*^87^, using effect size estimates and standard errors (option *SCHEME STDERR*). Results were clumped using the *clump_data* function of *TwoSampleMR*^88,89^ R package version 0.5.6) at an *r*^2^<0.001 based on the European 1000 Genomes Project reference panel with 10,000 kb window^90^. Results were then visualized in a Manhattan plot, which was constructed using the *gwaslab* Python package^91^.

### Mendelian randomization

Summary-level data sources for exposures, along with their descriptions, are provided in Supplementary Table 3. Two-sample MR was conducted using the *TwoSampleMR* package in R. The instrumental variable for each exposure was constructed by selecting SNVs from summary statistics files with a significant association (p < 5×10⁸), followed by clumping for linkage disequilibrium at an r^2^ < 0.001 threshold within a 10,000 kb window. For IL-6 receptor-mediated signaling activity, the genetic instrument was constructed as previously described^92^, using beta estimates derived from a cohort that excluded the UKB to minimize bias. Main association estimates were derived using random-effects IVW analysis. To account for potential bias due to horizontal pleiotropy, sensitivity analyses were performed using both MR-Egger and the weighted median estimator, as these methods are known to be more robust against pleiotropic effects^93,94^. Heterogeneity and horizontal pleiotropy of the genetic instruments were estimated using the *mr_heterogeneity* and *mr_pleiotropy_test* functions from the *TwoSampleMR* package. When MR-Egger indicated significant pleiotropy, we utilized the MR-PRESSO method from the *MRPRESSO*^95^ package in R to identify and exclude significant pleiotropic instrumental variables (P < 0.05), which were considered as “outliers”. Subsequently, IVW, weighted median, and MR-Egger analyses were carried out on the outlier-corrected models.

## Supplementary Material

Supplement 1

Supplement 2

## Figures and Tables

**Figure 1.**
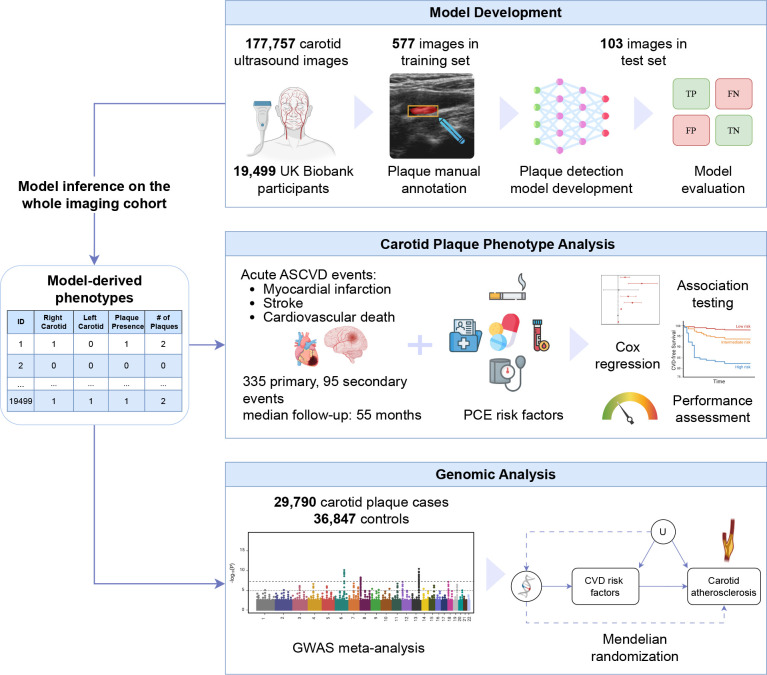
Summary of the study design. ASCVD – Atherosclerotic Cardiovascular Disease, CVD – Cardiovascular disease, PCE – Pooled Cohort Equatios, TP– true positive, FN – False Negative, FP – False Positive, TN – True Negative, GWAS – Genome-Wide Association Study

**Table 1.** Characteristics of the study cohort at the time of initial carotid ultrasound assessment.

	Total	Plaque absent	Plaque present	P-value for comparisons*
N	19,499	10,718	8,781	
Female, N (%)	9912 (50.8)	5688 (53.1)	4224 (48.1)	<0.001
Age, mean (SD)	64.6 (7.6)	63.4 (7.4)	66.1 (7.5)	<0.001
Smoking status, N (%)				<0.001
Never	12169 (62.4)	6951 (64.9)	5218 (59.4)	
Previous	6564 (33.7)	3384 (31.6)	3180 (36.2)	
Current	700 (3.6)	350 (3.3)	350 (4.0)	
Unknown	66 (0.3)	33 (0.3)	33 (0.4)	
SBP (mmHg), mean (SD)	138.6 (17.94)	137.18 (17.43)	140.42 (18.39)	<0.001
DBP (mmHg), mean (SD)	79.32 (9.78)	79.51 (9.75)	79.09 (9.81)	0.003
Total cholesterol (mmol/L), mean (SD)	5.72 (1.09)	5.70 (1.07)	5.74 (1.10)	0.009
LDL-C (mmol/L), mean (SD)	3.58 (0.83)	3.56 (0.82)	3.59 (0.84)	0.028
HDL-C (mmol/L), mean (SD)	1.48 (0.37)	1.47 (0.37)	1.48 (0.37)	0.708
HbA1c (mmol/mol), mean (SD)	35.13 (4.95)	34.91 (4.85)	35.40 (5.05)	<0.001
eGFR (mL/min/1.73m^2^), mean (SD)	93.58 (12.63)	94.52 (12.54)	92.42 (12.65)	<0.001
BMI (kg/m^2^), mean (SD)	26.60 (4.41)	26.80 (4.54)	26.37 (4.23)	<0.001
History of diabetes, N (%)	1073 (5.5)	511 (4.8)	562 (6.4)	<0.001
Statin usage, N (%)	4631 (23.7)	2131 (19.9)	2500 (28.5)	<0.001
Antihypertensive therapy, N (%)	4826 (24.7)	2312 (21.6)	2514 (28.6)	<0.001
Previous history of CVD, N (%)	1389 (7.1)	568 (5.3)	821 (9.3)	<0.001
Ethnicity, N (%)				<0.001
White	18932 (97.1)	10371 (96.8)	8561 (97.5)	
Asian	189.00 (1.0)	107 (1.0)	82 (0.9)	
Black	121.00 (0.6)	90 (0.8)	31 (0.4)	
Mixed	77.00 (0.3)	53 (0.5)	24 (0.3)	
Other/unknown	180.00 (0.9)	97 (0.9)	83 (0.9)	

SBP – Systolic Blood Pressure; DBP – Diastolic Blood Pressure; LDL-C – Low-Density Lipoprotein Cholesterol; HDL-C – High-Density Lipoprotein Cholesterol; HbA1c – Glycated Hemoglobin; eGFR – Estimated Glomerular Filtration Rate; BMI – Body Mass Index; CVD – Cardiovascular Disease.

* P-values were calculated using a two-sided t-test for continuous and a chi-square test for categorical variables.

**Table 2.** Metrics of discrimination, reclassification and overall model fit between Cox regression models with and without plaque information.

Model	Control / Cases	Log-likelihood ratio test p-value	cfNRI [95% CI] (p-value)	IDI [95 % CI] (p-value)	C-index [95% CI]	C-index difference (p-value)
CVD factors	19069 / 430	ref	ref	ref	0.745 [0.723; 0.767]	ref
CVD factors + Plaque presence		4.8×10^−4^	0.331 [0.217; 0.445] (1.26×10^−8^)	0.0022 [0.0002; 0.0050] (0.019)	0.747 [0.725; 0.769]	0.002 (0.222)
CVD factors + Plaque count		5.6×10^−4^	0.369 [0.260; 0.478] (2.97×10^−11^)	0.0024 [2×10^−5^; 0.0063] (0.048)	0.748 [0.726; 0.770]	0.003 (0.173)
CVD factors	17775 / 335	ref	ref	ref	0.744 [0.719; 0.770]	ref
CVD factors + Plaque presence		0.003	0.329 [0.206; 0.452] (1.65×10^−7^)	0.0019 [0.0003; 0.0052] (0.0079)	0.746 [0.721; 0.771]	0.002 (0.357)
CVD factors + Plaque count		0.006	0.330 [0.209; 0.450] (7.73×10^−8^)	0.0022 [0.0004; 0.0065] (0.0139)	0.746 [0.721; 0.772]	0.002 (0.349)
CVD factors	14283 / 234	ref	ref	ref	0.761 [0.732; 0.790]	ref
CVD factors + Plaque presence		0.014	0.308 [0.159; 0.457] (4.88×10^−5^)	0.0018 [0.0002; 0.0059] (0.014)	0.763 [0.734; 0.792]	0.002 (0.453)
CVD factors + Plaque count		0.039	0.308 [0.159; 0.458] (5.31×10^−5^)	0.0021 [0.0003; 0.0075] (0.0119)	0.763 [0.734; 0.792]	0.002 (0.471)

CVD – cardiovascular disease; cfNRI – category-free net reclassification improvement; C-index – concordance index; IDI – integrated discrimination improvement; ref – reference.

**Table 3.** Reclassification table showing the distribution of individuals who went on to develop or not major adverse cardiovascular events into two risk groups based on the recommended 7.5% threshold from PCE risk assessment, both with and without incorporating plaque information.

		PCE + Plaque presence		% reclassified	PCE + Plaque Count		% reclassified
	PCE	<7.5%	≥7.5%		<7.5%	≥7.5%	

Controls							
	<7.5%	16341	213	1	16300	254	2
	≥7.5%	90	485	16	82	493	14
Cases							
	<7.5%	250	17	6	247	20	7
	≥7.5%	4	47	8	4	47	8

Upward movement for cases indicates correct reclassification, while downward movement indicates incorrect reclassification. For controls, the opposite applies.

PCE – Pooled Cohort Equations; blue background indicates correctly reclassified individuals; orange background indicates wrongly reclassified individuals.

## Data Availability

The UKB provides an accessible research resource, available to researchers upon submitting a research proposal at https://www.ukbiobank.ac.uk. The GWAS meta-analysis data obtained in this study will be uploaded to the GWAS Catalog (https://www.ebi.ac.uk/gwas/home) upon the publication of the current manuscript. Additionally, the carotid plaque phenotypes derived from the developed model will be returned to UKB for use in future studies.
