# Supplementary material for: Deep Learning-Based Detection of Carotid Plaques Informs Cardiovascular Risk Prediction and Reveals Genetic Drivers of Atherosclerosis
Source: medRxiv. 2024 Oct 18:2024.10.17.24315675. Preprint. [Version 1] doi: 10.1101/2024.10.17.24315675 (PMC11527046; doi:10.1101/2024.10.17.24315675)

**Supplementary Figure 1.** Flowchart of the study participants and corresponding carotid ultrasound images.

**Supplementary Figure 2.** Results of the 5-fold cross-validation performed on a combined training and validation dataset. Model parameters were set as specified in the Methods section.

**
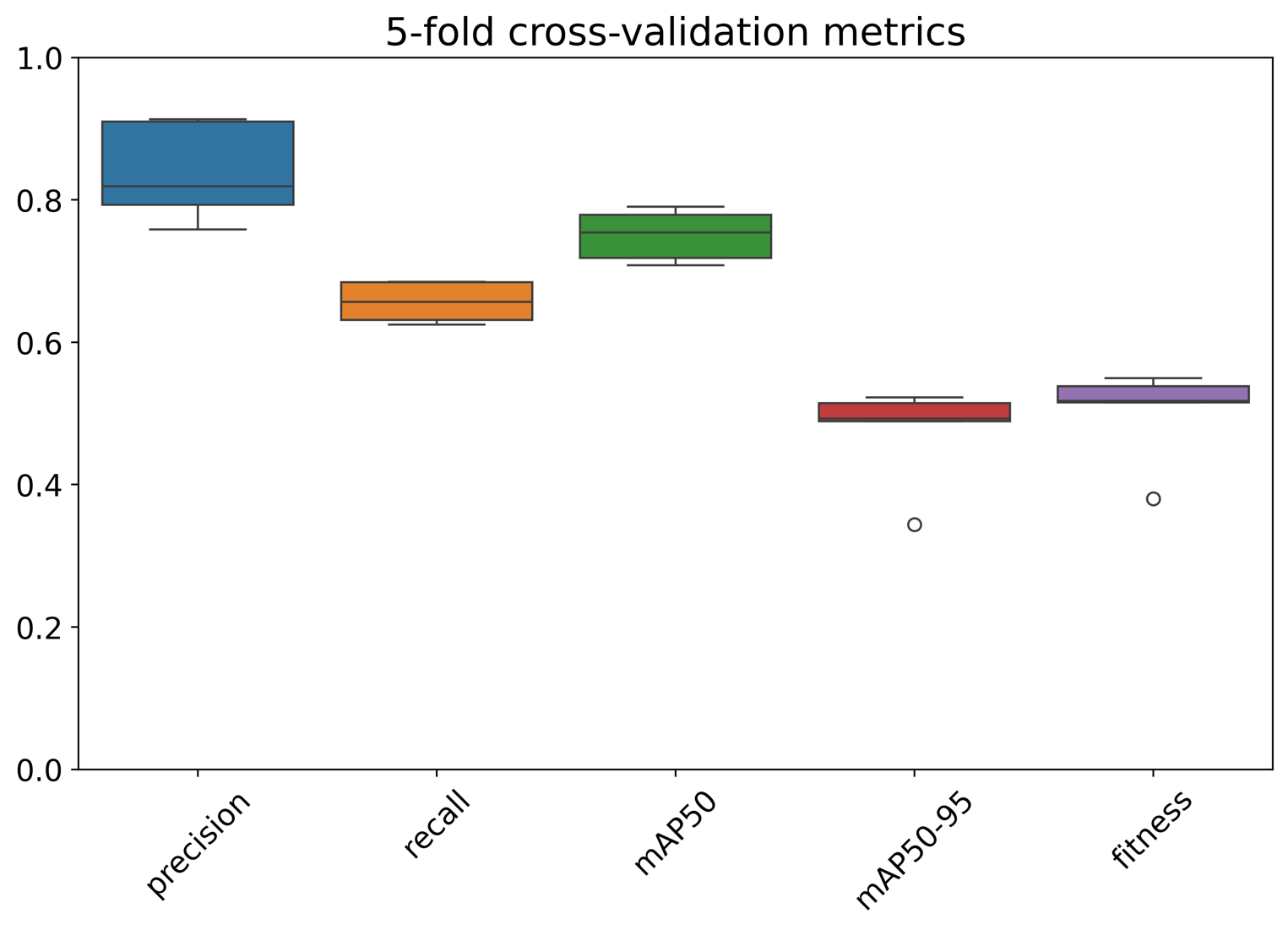
**

**Supplementary Figure 3.** Examples of the model’s predictions.

**Supplementary Figure 4.** Comparisons of three types of carotid intima-media thickness (IMT) measurements between individuals with model-predicted plaque and those without. **A)** Averaged measurements of both left and right carotid arteries; **B)** Left carotid artery; **C)** Right carotid artery

**Supplementary Figure 5.** Hazard ratios (HRs) from Cox models fitted on different subsamples for **A)** Major Adverse Cardiovascular Events (MACE) and its individual components: **B)** Myocardial infarction; **C)** Stroke. HR estimates for plaque presence and plaque number (one plaque, two or more plaques) were obtained using two different models for each subsample. All plaque-related variables were encoded as categorical.

**Supplementary Figure 6.** Calibration plots for Cox models fitted on different subsamples **A)** Major Adverse Cardiovascular Events (MACE) and its individual components: **B)** Myocardial infarction; **C)** Stroke.

**Supplementary Figure 7.** Calibration plots for the Pooled Cohort Equations (PCE) applied to the UKB cohort before and after recalibration and incorporating plaque information

**Supplementary Figure 8.** To categorize all the images according to their type, we wrote a Python script that counts the number of pixels in specific locations of the ultrasound machine picture frame, tracking the angle and type of examination. The obtained results were manually validated. The classified long axis images were cropped to a size of 480x448 to keep only the ultrasound image while maintaining the original resolution. In order to enhance contrast and decrease noise in the images, we applied two functions from the OpenCV library: median blur filtering (*ksize=5*) and Contrast Limited Adaptive Histogram Equalization (*clipLimit=2.0, tileGridSize=(8,8)*) respectively.


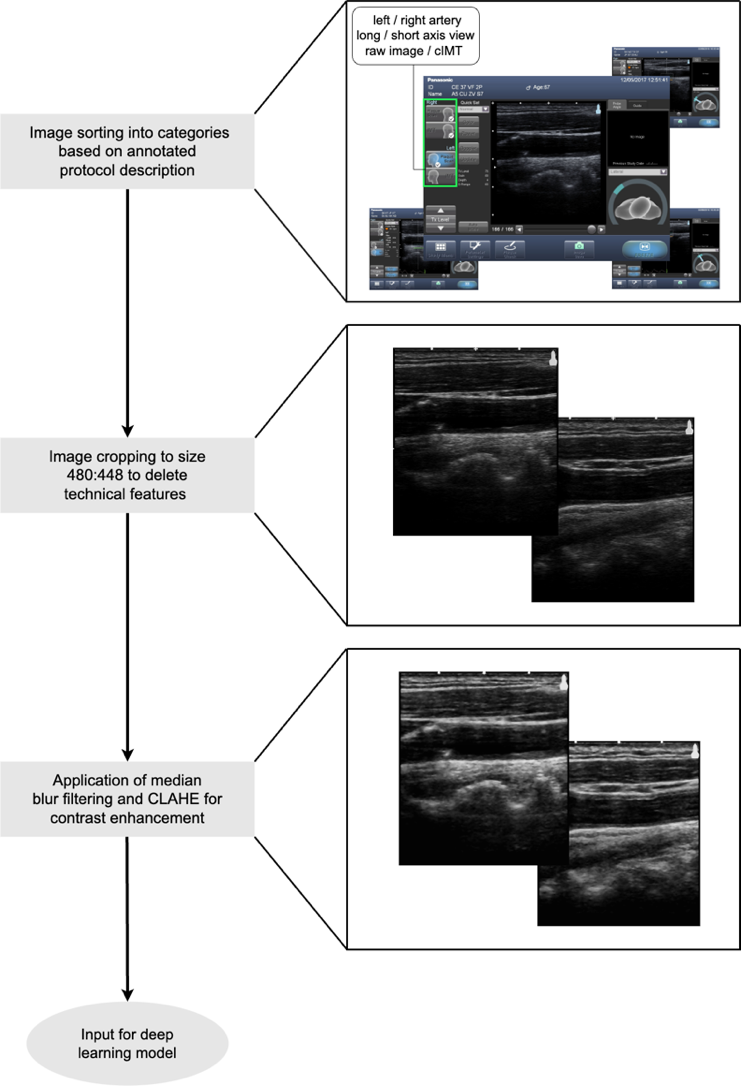

Supplement: Supplement 1 [file media-1.docx]
